# Hypertrophy of Adipose Tissues in Quail Embryos by *in ovo* Injection of All-*Trans* Retinoic Acid

**DOI:** 10.3389/fphys.2021.681562

**Published:** 2021-05-21

**Authors:** Dong-Hwan Kim, Joonbum Lee, Sanggu Kim, Hyun S. Lillehoj, Kichoon Lee

**Affiliations:** ^1^Department of Animal Sciences, The Ohio State University, Columbus, OH, United States; ^2^The Ohio State University Interdisciplinary Human Nutrition Program, The Ohio State University, Columbus, OH, United States; ^3^Department of Veterinary Biosciences, College of Veterinary Medicine, The Ohio State University, Columbus, OH, United States; ^4^Animal Bioscience and Biotechnology Laboratory, Beltsville Agricultural Research Center, Agricultural Research Service, United States Department of Agriculture, Beltsville, MD, United States

**Keywords:** all-*trans* retinoic acid, *in ovo* injection, adipocyte hypertrophy, fat accretion, embryonic development, quail

## Abstract

Excessive adipose accretion causes health issues in humans and decreases feed efficiency in poultry. Although vitamin A has been known to be involved in adipogenesis, effects of all-*trans* retinoic acid (atRA), as a metabolite of vitamin A, on embryonic adipose development have not been studied yet. Avian embryos are developing in confined egg environments, which can be directly modified to study effects of nutrients on embryonic adipogenesis. With the use of quail embryos, different concentrations of atRA (0 M to 10 μM) were injected *in ovo* at embryonic day (E) 9, and adipose tissues were sampled at E14. Percentages of fat pad weights in embryo weights were significantly increased in the group injected with 300 nM of atRA. Also, among three injection time points, E5, E7, or E9, E7 showed the most significant increase in weight and percentage of inguinal fat at E14. Injection of atRA at E7 increased fat cell size in E14 embryos with up-regulation of pro-adipogenic marker genes (*Pparγ* and *Fabp4*) and down-regulation of a preadipocyte marker gene (*Dlk1*) in adipose tissues. These data demonstrate that atRA promotes hypertrophic fat accretion in quail embryos, implying important roles of atRA in embryonic development of adipose tissues.

## Introduction

Excessive fat accretion causes obesity and associated diseases in humans. On the agricultural side, excessive fat is undesirable due to economic concerns of animal producers on low feed efficiency associated with fat accretion and health issues of consumers on high fat contents in meat. Discovery and understanding of roles of genetic and nutritional factors in fat regulation will lead to improving health and efficiency of animal production. Different from mammals, avian species depend on the various nutrient contents in the fertile egg for growth and development of embryos. During embryonic development of the chicken, adipose tissues begin to be visible from embryonic day (E) 10 and grown through hyperplasia and hypertrophy (Chen et al., 2014). Chicken embryos at E10–E12 have preadipocytes and multilocular immature fat cells (Chen et al., 2014). These cells are further developed toward more numbers of unilocular fat cells at E14 (Chen et al., 2014). This study showed how rapidly adipocytes are developed during embryonic development.

Vitamin A (retinol) is an essential nutrient in animals and involved in biological processes of adipose development. Retinol can be converted to all-*trans* retinoic acid (atRA), which functions as a key regulatory factor in embryonic development by controlling differentiation of many cell types including adipocytes. Previous studies showed that supplementation of high doses of atRA (over 1 μM) on 3T3-L1 cells blocked lipid accumulation (Mercader et al., 2007; Stoecker et al., 2017), but the supplementation of low concentration (10 nM) had pro-adipogenic effects on Ob1771 cells (Safonova et al., 1994). Our previous study also showed that atRA functions as a positive or negative regulator on adipogenic differentiation of 3T3-L1 cells by supplementation of mild or excessive doses, respectively (Kim et al., 2019). Levels of serum retinol were higher in obese children at the ages of 6–14 years (Aeberli et al., 2007). Supplementation of high levels of dietary vitamin A in obese rats significantly reduced body weight gain and adipose tissue weight (Jeyakumar et al., 2008). In addition, injection of atRA into mice reduced fat mass (Felipe et al., 2005; Mercader et al., 2006), but injection of retinol into new-born Angus cattle increased the ratio of intra-muscular fat and marbling score (Harris et al., 2018). However, roles of retinoids in fetal adipose development have not been reported. This might be due to potential difficulties in precisely modulating fetal retinoids such as an uncertain relationship of maternal retinoid levels with fetal levels or issues in direct delivery into fetus.

Avian species are suitable models for the research of developmental origins of health and disease because nutritional, hormonal, and chemical exposures can be strictly modulated by directly injecting exogenous factors into embryos at specific developmental stages, which cannot be easily accomplished in mammalian embryos. In poultry, adipogenic differentiation *in vitro* can be induced by supplementation of atRA (Kim et al., 2020a,b; Lee et al., 2021). Dietary supplementation of vitamin A during early post-hatch period increased fat accretion in broiler chickens (Savaris et al., 2020). The present study aims to investigate the cellular effects of atRA on adipose growth in quail embryos as an animal model.

## Materials and Methods

### Visualization of Embryonic Fat Pads

As described in our previous study (Chen et al., 2014), to visualize fat pads, quail embryo was removed from the egg, and feathers were removed by using forceps during washing with phosphate-buffered saline (PBS) three times. Then, to visualize the fat pads, the embryo was transferred into a 1% potassium hydroxide (KOH) solution after fixing in 70% ethanol because KOH, as a strong alkali, makes tissue soften, digest, and clear. After the skin was cleared, the embryos were visualized using a camera (EOS Rebel T7, Canon, Japan).

### *In ovo* Injection and Tissue Sampling

Experiments using poultry embryos are exempt from requiring University Institutional Animal Care and Use Committee approval, because avian embryos are not considered live animals by the Public Health Service Policy (ILAR News, 1991). All fertile eggs of Japanese quail were obtained from The Ohio State University poultry research farm. atRA (#R2625, Sigma-Aldrich, St. Louis, MO, United States) was diluted with dimethyl sulfoxide (DMSO). Before atRA was injected *in ovo*, the surface of fertile eggs that were confirmed by candling was cleaned with 70% ethanol. Eggshell at the two-thirds point of quail eggs was grinded by twisting with corner tip of blades (#4515, Ettore, Alameda, CA, United States) without disrupting the shell membrane. Two microliters of different concentrations of atRA (final concentrations: 100 nM to 10 μM) was injected through the shell membrane using a micropipette tip (#17000504, Rainin, Columbus, OH, United States), as shown in Figure 2A, at different embryonic ages (E5, E7, or E9) and then sealed with parafilm. The injected eggs were incubated at 37.5°C with 60% relative humidity until sampling inguinal fat pads at E12 or E14. DMSO was injected as a control (0 M).

### Histological Processing and Measurement of Fat Cell Size

Inguinal fat pads were 1fixed in 10% neutral buffered formalin for 48 h and then processed to embed in paraffin. Paraffin embedded fat tissues were cut into 5-μm slices. To measure fat cell size, serial sections of the embedded samples were de-paraffinized in xylene, re-hydrated in serial diluted ethanol, and then stained with hematoxylin and eosin (H&E). Stained slides were observed and imaged under a microscope (EVOS cell imaging system, Thermo Fisher Scientific, Waltham, MA, United States). ImageJ software (NIH ImageJ 1.52s) was used to determine fat cell size. The average of the adipocyte cross-sectional area was calculated by measuring randomly selected areas having large numbers of cells and dividing this by the total number of cells found within the area. At least 800 cells at E12 and 500 cells at E14 were evaluated per animal.

### Analysis of Gene Expression

After extraction of total RNA from inguinal fat tissues and cDNA synthesis by the methods described in our previous study (Kim et al., 2020a), quantitative PCR (qPCR) was performed in duplicate per sample to analyze gene expression associated with adipogenesis with specific primer sets; *Peroxisome proliferator-activated receptor gamma* (*Pparγ*, NCBI Reference Sequence: NM_001001460.1, F: 5′-GTGAATCTTGACCTGAATGATC AGGT, R: 5′-AGATTATCTTGTATATCTTCAATGGGCTTCA CAT), *fatty acid-binding protein 4* (*Fabp4*, NCBI Reference Sequence: XM_015855897.2, F: 5′-CAAGCTGGGTGAAGAGT TTGATG, R: 5′-CTCTTTTGCTGGTAACATTATTCATGGT GCA), and *Delta like non-canonical notch ligand 1* (*Dlk1*, NCBI Reference Sequence: XM_032445023.1, F: 5′-CTGCCATCT CAGGAAAGGACC, R: 5′-ACATGGGTTGGATTCACAGTC ATC). *Glyceraldehyde 3-phosphate dehydrogenase* (*Gapdh*, NCBI Reference Sequence: NM_204305.1, F: 5′-CTCTGTTGTTGAC CTGACCTG, R: 5′-CAAGTCCACAACACGGTTGCT) was used as a reference gene. The expression levels were normalized to those of genes by the 0 M group at E12, and all of the data were analyzed using the ddCt method (Livak and Schmittgen, 2001).

### Statistical Analysis

All data were expressed as means ± SEM. The data were analyzed by the Student or multiple *t*-test using GraphPad Prism software, version 6.02. *p* < 0.05 was considered as a statistically significant difference.

## Results

### Visualization of Developing Adipose Tissues at Different Embryonic Ages

Although adipose tissue in chicken embryos appears around E10 (Chen et al., 2014), embryonic ontogeny of adipose tissues in quail has not been reported yet. Therefore, to investigate the developmental stages of adipose growth during embryonic ages, ontogeny of embryonic adipose development was studied. When adipose tissues begin to appear, they are too small to be visible and distinguished from other tissues. Therefore, intact fat pads in the embryo were visualized using KOH that enables soft tissues transparent by alkaline hydrolysis of proteins and fat tissues distinguishably visible by saponification of lipids (Culling et al., 1974). This method can be applied for visualization of fat pads in various species.

**FIGURE 1:**
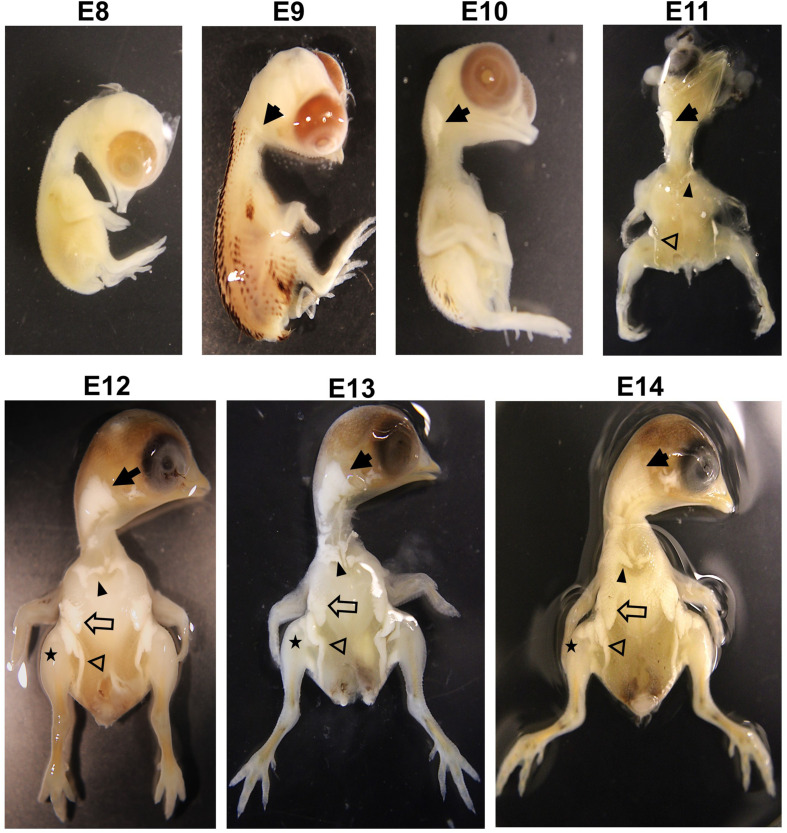
Visualization of fat pad on embryos in quail with 1% KOH at E8, E9, E10, E11, E12, E13, and E14. Solid arrow, neck fat; solid arrowhead, supraclavicular fat; opened arrow, breast fat; opened arrowhead, inguinal fat; star, leg fat. Images are not to scale.

The current study showed that adipose tissues in quail embryos were not found at E8 but initially appeared around the neck at E9 (Figure 1). Interestingly, the appearance of fat pads coincided with the time of feather development at E9 in quail and E10 in chickens (Hamburger and Hamilton, 1951; Chen et al., 2014; Nakamura et al., 2019), indicating that fat and feather development might initiate at similar developmental stages. At E10, fat pads around the neck were extended more but did not appear in other areas (Figure 1). In addition to neck fat pads, supraclavicular and inguinal fat pads first appeared at E11, and breast and leg fat pads were additionally shown from E12 (Figure 1).

### Effect of *in ovo* Injection With Different Concentrations of All-*Trans* Retinoic Acid on Adipose Weight

**FIGURE 2:**
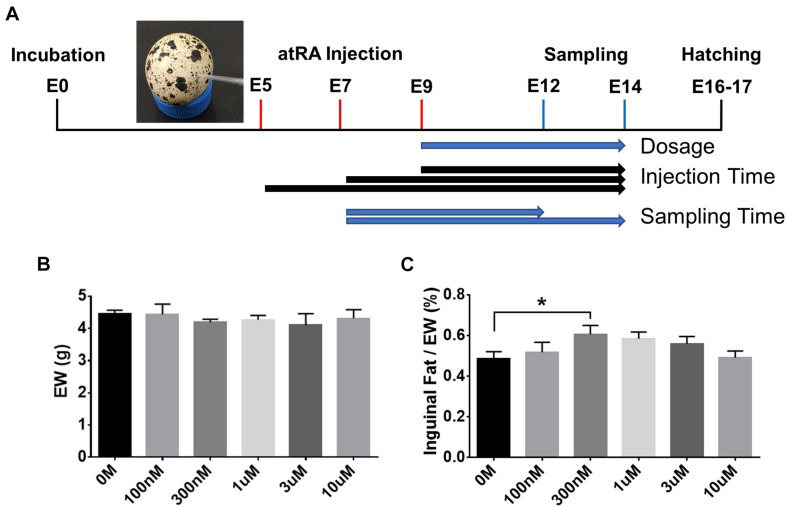
*In ovo* all-*trans* retinoic acid injection. **(A)** Schematic diagram of *in ovo* all-*trans* retinoic acid (atRA) injection. Quail eggs were injected with atRA at embryonic day (E) 5, E7, or E9 and sampled at E12 or E14. Embryo weight (EW, **B**) and percentages of inguinal fat weight in total EW **(C)**. Dose of 0 M, 100 nM, 300 nM, 1 μM, 3 μM, or 10 μM of atRA was injected *in ovo* at E9, and injected embryos were sampled at E14. *n* = 11, 7, 12, 12, 5, and 7 embryos for 0 M, 100 nM, 300 nM, 1 μM, 3 μM and 10 μM of atRA, respectively. All data were expressed as means ± SEM. The data were analyzed by multiple *t*-test using GraphPad Prism software, version 6.02. *p < 0.05.

Based on the ontogenetic data showing an appearance of adipose tissues in the neck area from E9, different dosages of atRA (final concentration: 0 M, 100 nM, 300 nM, 1 μM, 3 μM, or 10 μM) were injected into quail eggs at E9 to investigate the effect of atRA on embryonic development of adipose tissues (Figure 2A). Embryos injected with atRA were sampled at E14 to measure embryo weight (EW) and weight of inguinal fat tissues (Figure 2A). Although there was no difference in EW among all groups (Figure 2B), the percentages of weight of inguinal fat tissues in EW were gradually increased with increasing concentrations of atRA up to 300 nM and somewhat reduced at higher concentrations from 300 nM to 10 μM (Figure 2C). Especially, the group injected with 300 nM atRA showed a significantly increased percentage of inguinal fat tissue compared with those at the 0 M (1.25-fold, *p* < 0.05) (Figure 2C). Thus, the current data indicate that adipose growth can be influenced by atRA.

### Effect of *in ovo* Injection of All-*Trans* Retinoic Acid at Different Embryonic Ages on Adipose Weight

**FIGURE 3:**
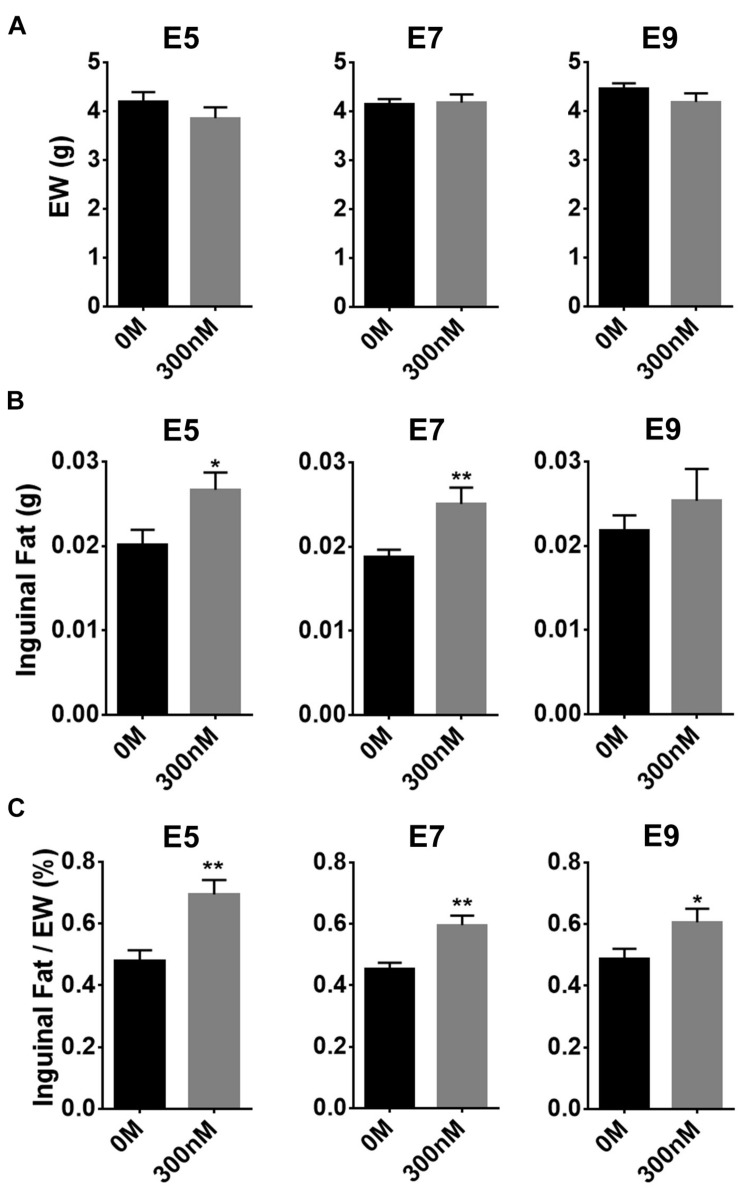
Effect of time of *in ovo* all-*trans* retinoic acid (atRA) injection. Embryo weight (EW) **(A)**, inguinal fat pad weights **(B)**, and percentages of weights of inguinal fat tissues in EW **(C)**. Both 0-M and 300-nM concentrations of atRA were injected *in ovo* at embryonic day (E) 5, E7, or E9; and injected embryos were sampled at E14. For 0 M or 300 nM of atRA, *n* = 8 and *n* = 8 embryos at E5, *n* = 9 and *n* = 8 embryos at E7, and *n* = 11 and *n* = 12 embryos at E9, respectively. All data were expressed as means ± SEM. The data were analyzed by a Student *t*-test using GraphPad Prism software, version 6.02. *p < 0.05, **p < 0.01.

To investigate to what extent atRA injection at three different ages, before (E5 or 7) and after (E9) appearance of adipose tissues, affects adipose growth, atRA was injected into eggs at those ages and adipose tissues were sampled at E14 (Figure 3). Weights of embryos at E14 were not affected by 300 nM of atRA injection at all three ages (Figure 3A). Although weights of inguinal fat pads were significantly increased by atRA injection at E5 (*p* < 0.05) and E7 (*p* < 0.01), percentages of inguinal fat weights in EW were significantly increased by injecting at all time points (E5: 1.45-fold, *p* < 0.01; E7: 1.32-fold, *p* < 0.01; E9: 1.25-fold, *p* < 0.05) (Figure 3C). These data demonstrated that injecting atRA before the appearance of fat pads is more effective in enhancing embryonic adipose growth.

### Hypertrophic Effect of All-*Trans* Retinoic Acid in Fat Tissues

**FIGURE 4:**
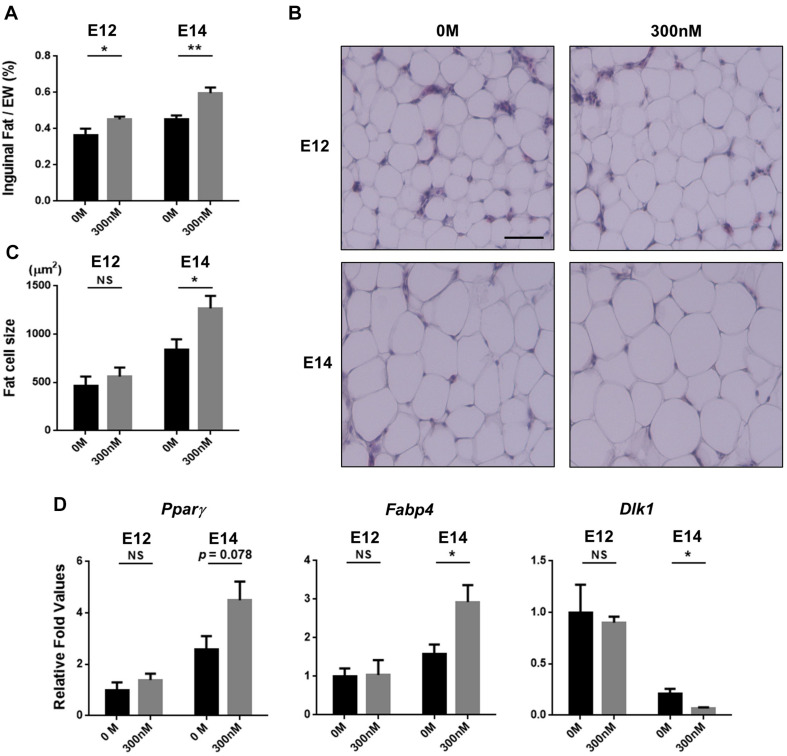
Hypertrophic fat cells by *in ovo* injection of all-*trans* retinoic acid (atRA). Percentages of inguinal fat weights in embryo weight (EW) **(A)**. Embryos were injected with 0 M or 300 nM of atRA at E7 and sampled at E12 or E14. For 0 M or 300 nM of atRA, *n* = 9 and *n* = 9 embryos at E12 and *n* = 9 and *n* = 8 embryos at E14, respectively. Scale bar: 100 μm. H&E stain of inguinal fat tissue **(B)** and comparisons of fat cell size **(C)**. Quantitative analysis of gene expression involved in adipogenesis by qPCR **(D)**. *n* = 4 for 0 M and 300 nM of atRA at E12 and E14, respectively. All data were expressed as means ± SEM. The data were analyzed by Student *t*-test using GraphPad Prism software, version 6.02. NS, no significance; *p < 0.05, **p < 0.01.

To determine whether the increased fat pad weights by atRA can be attributed to increasing cell size, embryos were sampled at E12 or E14 after injecting atRA at E7, and a histological examination was performed to measure sizes of fat cells. Injection of atRA at E7 resulted in increased percentages of inguinal fat weights in embryos at E12 and E14 (E12: 1.24-fold, *p* < 0.05; E14: 1.32-fold, *p* < 0.01) (Figure 4A). The sizes of fat cells at E12 were not significantly different between the two groups (0 M and 300 nM) (Figures 4B,C). In the samples that were injected with 300 nM of atRA at E7 and sampled at E14, size of inguinal fat cells was significantly increased compared with the 0 M (1.5-fold, *p* < 0.05) (Figures 4B,C). These data clearly showed that atRA injection resulted in increased fat pad weights and cell sizes during embryonic development in quail.

### Expression Levels of Genes Involved in Adipogenesis

To further investigate effects of atRA on expression of adipogenic marker genes in embryonic days, expression levels of marker genes for preadipocytes (*Dlk1*) and adipocytes (*Pparγ* and *Fabp4*) were analyzed. Injection of 300 nM atRA at E7 enhanced expression levels of *Pparγ* in E14 inguinal adipose tissues by 75% compared with the 0 M, although the difference was not statistically significant (*p* = 0.078) (Figure 4D). Expression of *Fabp4* in E14 adipose tissues was significantly up-regulated (85%, *p* < 0.05) by the atRA injection at E7 compared with the 0 M (Figure 4D). In addition, atRA injection at E7 significantly down-regulated expression levels of *Dlk1* by 68% (*p* < 0.05) in the adipose tissues at E14 compared with the 0 M (Figure 4D). These results indicate that *in ovo* injection of atRA caused enhanced expression of adipogenic markers and reduced expression of the preadipocyte marker, suggesting promotion of embryonic adipogenesis by injecting atRA *in ovo* in quail.

## Discussion

Avian species, as oviparous animals, have embryonic development within a unique egg environment where nutritional and hormonal factors can be directly injected to study the effect of these factors on processes of embryonic adipogenesis. In addition, different stages of embryos can be obtained from the eggs. However, it is difficult to directly modulate nutritional and hormonal factors in mammalian embryos and to study embryonic development without scarifying dams. For these reasons, chicken embryos have been used to visually aid in presenting embryonic development of adipose tissues (Chen et al., 2014) and to study a nutritional factor, selenium, in adipogenic differentiation by injecting *in ovo* (Hassan et al., 2014). In addition, the current study is the first demonstration that shows the visual evidence of fast-growing adipose tissues during quail embryonic development. Taken together, avian species are suitable models to study for embryonic adipose growth.

Vitamin A and its metabolites such as atRA have been used for the study of adipogenic differentiation in animals. Our previous study (Kim et al., 2019) showed different effects of atRA on differentiation of 3T3-L1 cells depending on atRA concentrations; atRA showed a pro-adipogenic effect at low concentrations but an anti-adipogenic effect at high concentrations. The current study shows the bell-shaped distribution of percentages of fat pad weights in EW with increasing concentrations (0–10 μM) of atRA (Figure 2C). The highest fat percentage was shown at 300 nM, and increased fat percentages were gradually diminished by injecting over 300 nM of atRA. These data are somewhat consistent with the previous study by showing anti-adipogenic activities of high concentrations of atRA *in vitro* (Muenzner et al., 2013) and reduced size of fat cells by high levels of atRA administration *in vivo* (Mercader et al., 2006).

The previous study showed that injection of atRA resulted in reduction of adipose tissues in adult mice (Amengual et al., 2010). In newborn Angers calves, injection of vitamin A significantly increased percentages of intramuscular fat (Harris et al., 2018). In broiler chickens, fat deposition rate was increased during 1–21 days after hatching but decreased during 22–42 days by dietary supplementation of vitamin A (Savaris et al., 2020). In the current study, *in ovo* injection of 300 nM atRA at E5–E7 resulted in an increase of weights and percentages of adipose tissues (Figure 3). The previous and current studies show that atRA can positively or negatively affect fat deposition in animals. In general, rate of fat accretion in adult animals is determined by a balance of lipolysis and lipogenesis (Cornelius, 1994). However, because avian embryos constantly receive required nutrients from the confined egg environments (Kimura and Warshaw, 1983), both adipocyte differentiation and lipogenesis mainly contribute to growth of embryonic adipose tissues until hatching when lipolysis starts to be active to provide energy for breaking the eggshell (Chen et al., 2014). In the current study, because embryos injected with atRA were sampled at E14 approximately 3 days before hatching in quail, increased fat weights with adipocyte hypertrophy in quail embryos by atRA might be attributed from enhanced lipogenesis rather than changes in lipolysis.

Our previous *in vitro* studies showed that atRA promoted differentiation of murine preadipocyte cell line (3T3-L1) into adipocytes (Kim et al., 2019) and induced adipogenic differentiation of chicken embryonic fibroblast (CEF) cell line (Lee et al., 2021) and primary CEFs (Kim et al., 2020a). These pro-adipogenic roles of atRA were also demonstrated by the finding that it increased lipid accumulation by atRA treatment *in vitro*. With the support of these *in vitro* studies, *in ovo* injection of atRA in the current study resulted in adipose hypertrophy. Interestingly, our previous study with a knockout mouse model demonstrated pro-adipogenic function of retinoids by the finding that mice with knockout of the Raldh1 gene, encoding an enzyme producing atRA from retinaldehyde, are lean with less fat (Yasmeen et al., 2013). In addition, Raldh1-deficient cells have impaired adipogenesis *in vitro*, which is rescued by RA supplementation (Reichert et al., 2011). Furthermore, atRA enhanced fat accumulation in zebra fish embryos (Fraher et al., 2015). These previous and current studies support the positive roles of atRA in adipocyte differentiation and fat accretion.

As *Pparγ* and *Fabp4* have been used as well-known adipogenic markers, up-regulation of the two genes suggests promotion of adipocyte differentiation in embryonic adipose tissues by atRA. In general, differentiation potential is negatively correlated with proliferative activities. It is possible that promotion of adipocyte differentiation by atRA may reduce proliferation potential and, consequently, reduce population of preadipocytes in embryonic adipose tissues. In this regard, it is interesting to relate adipogenic potential with population of preadipocytes by assessing a reliable preadipocyte maker. Dlk1, also known as Pref-1, was originally discovered by comparing expression profiles before and after differentiation of 3T3-L1 preadipocytes and identifying Dlk1 as a predominantly expressed gene in preadipocytes (Smas and Sul, 1993). Our previous *in vivo* studies demonstrated anti-adipogenic function of Dlk1 through findings that reduced fat in Dlk1 overexpressed mice (Lee et al., 2003) and obesity phenotype in Dlk1 knockout mice (Moon et al., 2002). Therefore, Dlk1 has been used as a preadipocyte marker in humans (Krautgasser et al., 2019), pigs (Ahn et al., 2013), cattle (Cai et al., 2018), chickens (Shin et al., 2008), and quail (Ahn et al., 2015). In the current study, increased cell size in inguinal adipose tissues with increasing age (E12–E14) is accompanied with up-regulation of *Pparγ* and *Fabp4* and down-regulation of *Dlk1* expression regardless of treatment groups (Figure 4D), indicating temporal progresses of adipogenic differentiation during embryonic development in quail. In addition to up-regulation of *Pparγ* and *Fabp4*, a lesser expression of *Dlk1* by 300 nM atRA compared with the control (0 M) at E14 suggests that adipocyte differentiation might be promoted by atRA and the preadipocyte population might be consequently reduced. In agreement with the current study, supplementation of atRA in 3D cultures of subcutaneous primary human preadipocytes contributed to adipocyte hypertrophy by regulating transcriptional factors involved in adipogenesis (Pope et al., 2020). For these reasons, it is possible that promotion of preadipocyte differentiation by atRA can contribute to increased fat mass in quail embryos. These findings support pro-adipogenic function of atRA during embryonic development.

Our finding showed that *in ovo* injection of 300 nM of atRA resulted in increased fat pad weights and fat cell size. Although the current study supports pro-adipogenic function of atRA in embryos, effects of dietary vitamin A or administration of RA into animals on fat accretion might be variable depending on concentrations of retinoids and ages of animals. Therefore, results from *in vivo* studies with retinoids should be carefully interpreted with consideration of these factors. Our experimental approach with *in ovo* injection in avian species can be applied to investigate effects of nutritional, hormonal, and pharmaceutical factors on embryonic development of various tissues and organs.

## Data Availability Statement

The original contributions presented in the study are included in the article/supplementary material, further inquiries can be directed to the corresponding author/s.

## Ethics Statement

Ethical review and approval was not required for the animal study because Experiments using poultry embryos are exempt from requiring University Institutional Animal Care and Use Committee approval, because avian embryos are not considered live animals by the Public Health Service Policy (ILAR News, 1991).

## Author Contributions

KL: conceptualization and supervision. D-HK: data curation and visualization. D-HK, JL, SK, and HL: formal analysis. D-HK, JL, and KL: investigation. D-HK and JL: methodology and writing—original draft. KL and HL: writing—review and editing. All authors have read and agreed to the published version of the manuscript.

## Conflict of Interest

The authors declare that the research was conducted in the absence of any commercial or financial relationships that could be construed as a potential conflict of interest.
